# CAGEfightR: analysis of 5′-end data using R/Bioconductor

**DOI:** 10.1186/s12859-019-3029-5

**Published:** 2019-10-04

**Authors:** Malte Thodberg, Axel Thieffry, Kristoffer Vitting-Seerup, Robin Andersson, Albin Sandelin

**Affiliations:** 1 Department of Biology, University of Copenhagen, Ole Maaløes Vej 5, DK2100, Copenhagen N, Denmark; 2 Biotech Research and Innovation Centre, University of Copenhagen, Ole Maaløes Vej 5, DK2100, Copenhagen N, Denmark; 3 Danish Cancer Society, Strandboulevarden 49 DK2100, Copenhagen Ø, Denmark

**Keywords:** Transcription start site, Promoter, Enhancer, Enhancer RNA, CAGE, 5′-end methods, R-package, Bioconductor

## Abstract

**Background:**

5′-end sequencing assays, and Cap Analysis of Gene Expression (CAGE) in particular, have been instrumental in studying transcriptional regulation. 5′-end methods provide genome-wide maps of transcription start sites (TSSs) with base pair resolution. Because active enhancers often feature bidirectional TSSs, such data can also be used to predict enhancer candidates. The current availability of mature and comprehensive computational tools for the analysis of 5′-end data is limited, preventing efficient analysis of new and existing 5′-end data.

**Results:**

We present CAGEfightR, a framework for analysis of CAGE and other 5′-end data implemented as an R/Bioconductor-package. CAGEfightR can import data from BigWig files and allows for fast and memory efficient prediction and analysis of TSSs and enhancers. Downstream analyses include quantification, normalization, annotation with transcript and gene models, TSS shape statistics, linking TSSs to enhancers via co-expression, identification of enhancer clusters, and genome-browser style visualization. While built to analyze CAGE data, we demonstrate the utility of CAGEfightR in analyzing nascent RNA 5′-data (PRO-Cap). CAGEfightR is implemented using standard Bioconductor classes, making it easy to learn, use and combine with other Bioconductor packages, for example popular differential expression tools such as limma, DESeq2 and edgeR.

**Conclusions:**

CAGEfightR provides a single, scalable and easy-to-use framework for comprehensive downstream analysis of 5′-end data. CAGEfightR is designed to be interoperable with other Bioconductor packages, thereby unlocking hundreds of mature transcriptomic analysis tools for 5′-end data. CAGEfightR is freely available via Bioconductor: *bioconductor.org/packages/CAGEfightR**.*

**Electronic supplementary material:**

The online version of this article (10.1186/s12859-019-3029-5) contains supplementary material, which is available to authorized users.

## Background

Transcription start sites (TSSs) are central entities of transcriptional regulation, where a wide range of cues from surrounding factors such as core promoter elements, transcription factor binding sites, chromatin modifications, and distal elements such as enhancers and silencers are integrated to decide whether transcription initiation takes place, and with what rate [1–3]. Hence, accurate identification of TSSs and their activity is a prerequisite for understanding gene regulation.

Several genome-wide, high-throughput sequencing assays have been developed for identifying TSS activity, all based on the idea of capturing and sequencing only the 5′-end of RNAs (called tags), leading to the collective name of 5′-end methods [4]. In terms of TSS identification, such methods have distinct advantages over other assays, e.g. RNA-sequencing (RNA-Seq) and Chromatin Immunoprecipitation Sequencing (ChIP-Seq). While RNA-Seq is widely used for studying gene expression and splicing, it is ineffective for accurate detection of TSSs. This is due to the random fragmentation of RNA molecules, leading to a trail-off of sequencing reads near the end of transcripts. In contrast, 5′-end methods effectively pile up reads at TSSs, providing high local coverage for accurate prediction of TSSs. Similarly, ChIP-Seq targeting RNA polymerase II or pre-initiation complex proteins has low positional resolution due to the length of ChIP-Seq fragments, and does not explicitly measure TSS usage.

The majority of available 5′-end methods capture steady-state capped RNAs (Table 1). This allows for identification of messenger RNA (mRNA) TSSs, including non-characterized alternative TSSs since the methods are not contingent on annotated transcript models. Alternative TSS usage is often tissue/cell-specific, and common in mammals [14, 15], plants [16], insects [17] and fungi [18, 19]. TSSs of long non-coding RNA (lncRNAs) can be detected and quantified in a similar fashion, often with greater precision than RNA-Seq alone [20]. Enhancer RNAs (eRNAs) is a class of non-coding RNAs which has attracted considerable interest, since they are transcribed from active enhancer regions, making it possible to predict enhancers using 5′-end data [21]. A set of 5′-end methods (Table 1) have been developed specifically for capturing nascent capped RNA to measure transcription as opposed to steady-state RNA levels, thereby enriching for unstable RNAs [12], including eRNAs.

**Table 1:** Examples of popular 5′-end methods

Technology	Acronym	RNA state	Ref.
Cap Analysis of Gene Expression	CAGE	Steady-tate	[5]
Nano CAGE	NanoCAGE	Steady-state	[6]
Super Low Input Carrier CAGE	SLIC-CAGE	Steady-state	[7]
no-Amplification non-Tagging CAGE	nAnT-iCAGE	Steady-state	[8]
Transcription Start Site Sequencing	TSS-Seq	Steady-state	[9]
RNA Annotation and Mapping of Promoters for the Analysis of Gene Expression	RAMPAGE	Steady-state	[10]
Single-cell Tagged Reverse transcription	STRT	Steady-state	[11]
Precision Nuclear Run-on Sequencing for RNA Polymerase II Start Sites	PRO-Cap	Nascent	[12]
5′ Global Run-on Sequencing	GRO-Cap/5′ GRO-Seq	Nascent	[13]

Cap Analysis of Gene Expression (CAGE [5]), based on reverse transcription of total RNA followed by cap-trapping, is arguably the most used 5′-end method and has the widest range of developed protocols (Table 1). CAGE has been applied in a multitude of different settings, including consortiums (FANTOM [15] and ENCODE [22]), multiple species (mammals [14], insects [17, 23, 24], fungi [18, 19], plants [6], etc.) and in clinical settings (inflammatory bowel disease [25], diabetes [26], cancer [27], Retts Syndrome [28]). Despite its wide usage, the current toolbase available for analyzing 5′-end data is not as developed as that for RNA-Seq or ChIP-Seq (Table 2). Most tools are either stand-alone pipelines (MOIRAI [29], RECLU [32], etc.) or focused on a single analysis problem, e.g. tag clustering (paraclu [30], CapFilter [6], etc., further discussed below), making it hard to combine different tools. An alternative to stand-alone tools is using R-packages from the Bioconductor [39] project, which allows easier interoperability between tools due to shared data representations. Bioconductor currently contains three packages (CAGEr [34], icetea [37], TSRchitect [38]) for analyzing 5′-end data in general and CAGE in particular. While these packages offer functionality for TSS identification, quantification and annotation, they lack any functions for predicting, quantifying and analyzing enhancer candidates, and are not efficiently scalable for large datasets.

**Table 2:** Examples of software packages for analyzing 5′-end data (including CAGE)

Tool	Implementation	Input data	Tag Clustering	TSS candidate shape	Differential Expression	Gene-level analysis	Unique features
MOIRAI [29]	Graphical User Interface	FASTQ	paraclu [30]	None	None inbuilt	None	rRNAdust, TagDust [31]
RECLU [32]	Bash	BED	modified paraclu [30]	None	edgeR [33]	None	Hierarchical TSSs
CAGEr [34]	R/Bioconductor	BAM	distance or paraclu [30]	IQR	DESeq2 [35]	Gene expression	G-bias correction [36], power-law normalization, TSS shifts
icetea [37]	R/Bioconductor	FASTQ	Sliding window	None	edgeR [33]	Gene expression	Paired-end methods, mapping via R
TSRchitect [38]	R/Bioconductor	BAM	X-means	Shape Index [17]	None inbuilt	None	Paired-end methods
CAGEfightR	R/Bioconductor	BigWig	Slice-reduce	IQR, entropy, etc.	None inbuilt	Gene expression and alternative TSS usage	Enhancer calling, TSS-enhancer co-expression, super enhancers

To solve the above problems, we here introduce the CAGEfightR R/Bioconductor package for analyzing 5′-end data. CAGEfightR is the first single framework that robustly detects, quantifies, annotates, links and visualizes TSSs and enhancer candidates in a manner that is highly compatible with other Bioconductor packages. The memory efficient and scalable implementation allows CAGEfightR to be used on datasets ranging from small-scale experiments to consortia-level projects. In this way, CAGEfightR unlocks hundreds of packages developed for RNA-Seq and ChIP-Seq for CAGE and similar types of 5′-end data.

## Implementation

CAGEfightR is implemented purely in R making use of several R-packages from the Bioconductor project. It is based on standard Bioconductor S4-classes, primarily GRanges (GenomicRanges), RangedSummarizedExperiment (SummarizedExperiment) [40] and GInteractions (InteractionSet) [41] and visualization via Gviz [42] and GenomicInteractions [43]. This makes it easy to use CAGEfightR in conjunction with other Bioconductor packages.

5′-end data is conventionally stored, shared and analyzed by first mapping tags to the genome, followed by counting the number of 5′-ends of tags mapping to each individual base pair (bp), on each strand. In CAGE terminology, such data are referred to as CAGE-defined TSSs (CTSSs) [36], but we use the term generally for all 5′-end methods here. The processing of tags differs between 5′-methods due to distinctive protocols (5′-end isolation technique, single-end vs. paired-end sequencing, etc.), and for CAGE in particular specialized tools have been developed, e.g. rRNAdust for removing contaminant ribosomal RNA (http://fantom.gsc.riken.jp/5/suppl/rRNAdust/) and/or removing G’s added by reverse transcriptase at cDNA 5′-ends [36]. While filtering, mapping, and counting of tags can be done efficiently by dedicated tools a single library at a time, CAGEfightR is focused on analysis from the point when multiple libraries are jointly analyzed. To be as general as possible, CAGEfightR was designed to import and analyze 5′-end data after mapping and processing by starting from CTSSs from each library stored as BigWig files.

Most genome bps are not CTSSs (have no tags mapped to them), and only a small fraction of CTSSs have a high number of tags. CAGEfightR takes advantage of this sparsity by using sparse representations to efficiently store and analyze large CTSS datasets using little memory. This allows tens of samples to be analyzed on a typical laptop computer and hundreds of samples on a typical server. Most computationally heavy tasks can be parallelized, providing further speed increases when multiple cores or clusters are available.

**Fig. 1:**
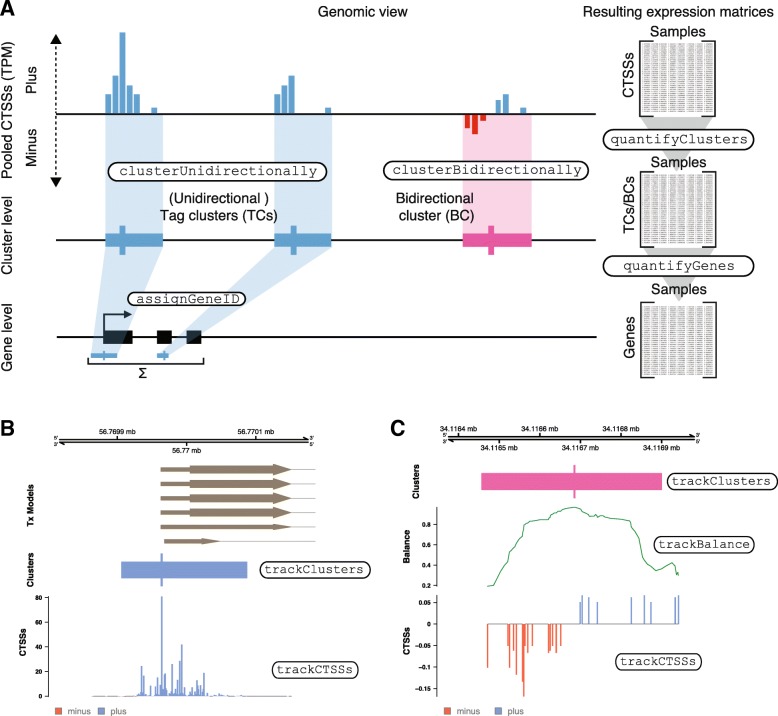
Introduction to CAGEfightR. **a**: Overview of CAGEfightR analysis steps: CAGEfightR can import CTSSs (the number of tag 5′-ends mapping to each bp position) and calculate a pooled CTSSs signal across all samples (top). The pooled CTSSs signal on the same strand can be used to identify unidirectional or Tag Clusters (TCs) which corresponds to groups of nearby TSSs or bidirectional clusters (BCs) which are candidate enhancers (middle). TCs can furthermore be assigned to genes using annotated gene models and summed to provide an estimate of gene expression (bottom). Each of these levels of analysis is associated with an expression matrix (right). The names of used CAGEfightR functions for respective analyses are highlighted. **b**: Example of unidirectional clustering. The bottom track shows the pooled CTSS signal (pooled TPM) at each bp along the genome. Middle track shows a Tag Cluster (TC) based on the CTSS data below as a block, where the position with the highest pooled CTSS signal is indicated (TC peak). The top track shows UCSC transcripts models (lines/thin blocks/thick blocks are intronic/UTR/CDS regions, respectively). **c**: Example of bidirectional clustering to predict enhancers. Bottom track shows pooled CTSS signal as in panel B, but with signal on both strands (red, negative bars indicate minus strand and blue, positive values indicate plus strand). The middle track shows the balance score (Bhattacharyya coefficient, Additional file 1 :Figure S1A) calculated along the genomic region. Top track shows the resulting Bidirectional Cluster (BC) as a block in pink indicating lack of strand information, where the single bp with the highest balance score is indicated

As described below, CAGEfightR can analyze 5′-end data on three different levels: bp-accurate CTSSs (Fig. 1a, top), clusters of nearby CTSSs (Fig. 1a, middle) or expression summed over known gene models (Fig. 1a, bottom), where each analysis level is associated with a specific expression matrix (Fig. 1a, right). These expression matrices and other data structures used in CAGEfightR are designed to be readily usable by other Bioconductor packages, in particular popular differential expression packages such as limma [44], edgeR [33], DESeq2 [35], DEXSeq [45], DRIMSeq [46], etc.

## Results and discussion

**Fig. 2:**
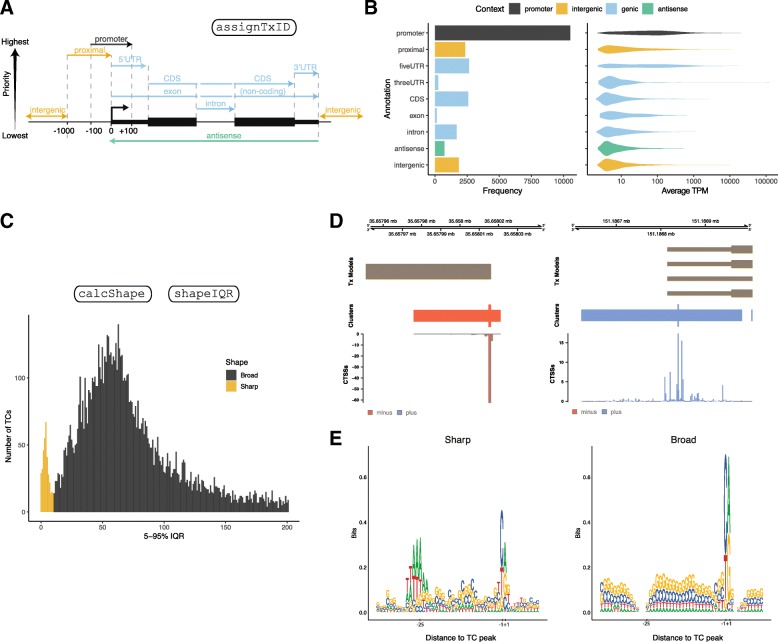
Analysis of tag clusters. **a**: Schematic of the hierarchical annotation scheme used by CAGEfightR (bp distances are modifiable by the user). Categories towards the top have higher priority when assigning clusters to their transcript-model context. **b**: Annotation of TCs from the Hela set. Y-axis shows the 9 annotation categories defined in panel A. Left bar plot shows the number of TCs falling into each category. Violin plot to the right shows the average pooled expression (log_10_-scaled TPM). Color indicates the overall context within gene models. **c**: Distribution of the 5–95% IQR for TCs from the Hela set. Color indicates the threshold (IQR = 10) to define sharp and broad classes. **d**: Examples of sharp and broad class TCs from panel C. Genome-browser style visualization as in Fig. 1b-c. Left panel shows a sharp CTSS distribution, right panel shows a broad CTSS distribution. **e**: Core promoter sequence patterns of sharp and broad classes of TCs from panel C. Y-axis shows information content as bits. X-axis shows genomic position relative to TC peak

**Fig. 3:**
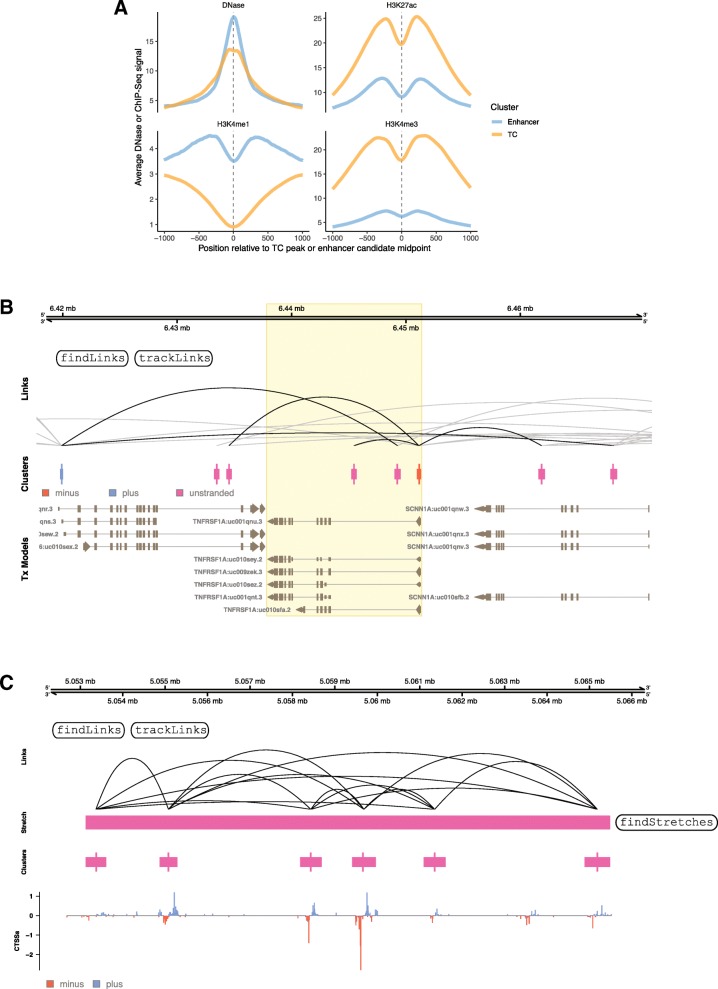
Analysis of enhancer candidates. **a:** Chromatin modifications at TCs and enhancer candidates from the Hela set. X-axis shows distance to TC peak or enhancer candidate midpoint. Y-axis are average signal of respective DNase-Seq or ChIP-Seq data in the given panel. Color indicates whether signals are centered on TC peaks or enhancer candidate midpoints. **b:** Example of predicted enhancer candidate-TC links in the ulcerative colitis set. Plot shows a genome-browser style visualization of correlations between TCs and enhancer candidates around the TNFRSF1A gene (central group of transcripts, highlighted). Bottom track shows UCSC transcript models and middle track shows clusters, as in Fig. 1b-c. Top track shows predicted TC-enhancer candidate links, where higher arches correspond to more significant correlations. Light grey links indicate that only one part of the pair is within the visualized region. **c:** Example of predicted enhancer stretch in the ulcerative colitis set. Bottom track shows UCSC transcript models and lower-middle track shows clusters, as in Fig. 1b-c. Upper-middle track shows the identified stretch of enhancer candidates. Top track shows correlations between enhancer candidates as in B

**Fig. 4:**
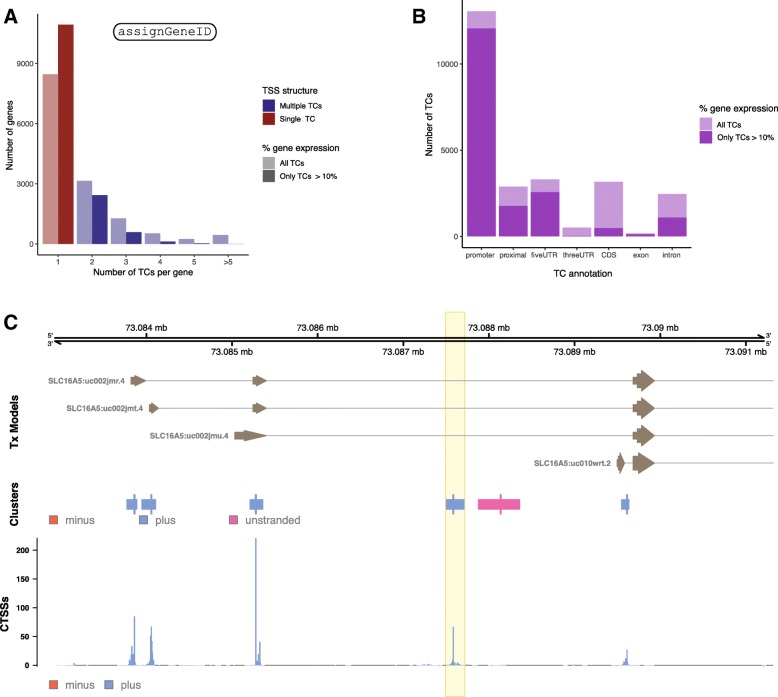
Analysis of alternative TSSs. **a**: Number of alternative TSS candidates per gene, as identified by TCs. X-axis shows number of TCs per gene. Y-axis shows the number of genes. Color indicates genes showing alternative TSS usage, having either a single or multiple TCs. Transparency indicates numbers before/after filtering away TCs making up less than 10% of total gene expression. **b**: Transcript context of TCs within genes. X-axis shows annotation categories from Fig. 1b. Y-Axis shows the number of TCs within each category. Transparency indicates whether the TC contributes > 10% of total gene expression. **c**: Genome-browser style example of an unannotated TC in the SLC16A5 gene. Organized as Fig. 1b-c. The novel alternative intronic TC is highlighted, found next to an intronic enhancer candidate

**Fig. 5:**
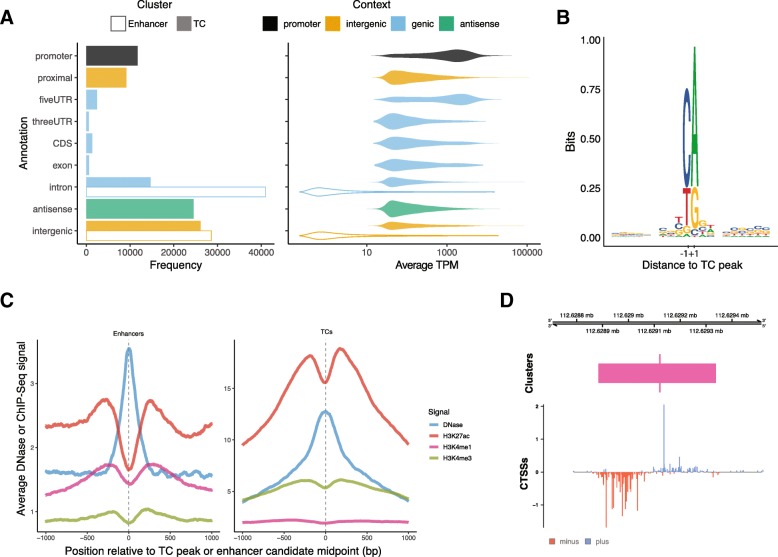
Analysis of PRO-Cap data. **a**: Annotation of TCs and enhancer candidates from the PRO-Cap set. Organized as Fig. 2b, but with enhancer candidates added, as indicated by transparency. **b**: Sequence logo of the core promoter sequence around TCs aligned on TC peaks. **c**: Chromatin modifications at TCs and enhancer candidates from the PRO-Cap set. Average signal of ChIP-Seq or DNase-Seq of 10,000 randomly sampled enhancer (left) and TCs (right). X-axis indicates distance to TC peak or enhancer candidate midpoint. Y-axes shows average ChIP-Seq or DNase-Seq signal as indicated by color. **d**: Example of intergenic enhancer candidate from the PRO-Cap set. Figure organised as Fig. 1b-c

Below, we overview the core functionality of CAGEfightR with examples using previously published 5′-end data. Names of the main CAGEfightR functions for each analysis are indicated in Fig. 1, 2, 3, 4, 5. Genome-browser figures (Figs. 1b-c, 2d, 3b-c, 4c, 5d) are otherwise unedited output from R/CAGEfightR.

### Analysis of 5′-end tags

CAGEfightR can import CTSSs from BigWig files and quantify their expression levels across all samples. The CTSSs can then be normalized to Tags-Per-Million (TPM) and summed across samples to yield a global or pooled CTSS signal (Fig. 1a, top). In case of a large number and/or low quality samples, CAGEfightR offers various strategies for calculating more robust pooled CTSSs signals, chiefly by filtering CTSSs only observed in a single or few samples. The pooled CTSS signal can be visualized in genome-browser style along the genome (Fig. 1b-c, 2d, 3c, 4c, 5d).

### Analysis of tag clusters

Pooled CTSSs can be used to identify clusters of closely spaced CTSSs on the same strand, referred to as unidirectional clusters, or conventionally Tag Clusters (TCs) in most CAGE papers. CAGEfightR identifies TCs using a slice-reduce approach: First, CTSSs with pooled CTSS signal below a chosen threshold are discarded (slice) and surviving CTSSs on the same strand are then merged into clusters (reduce) (Fig. 1a-b). CAGEfightR includes a host of functions for analyzing such TCs, including filtering on expression, hierarchical annotation of TCs with transcript models and analysis of TC shapes (see below).

TCs reflect the fact that when RNA-polymerase associates with the DNA, it rarely initiates from a single bp, but rather from an array of nearby bps. Such arrays are expected to produce nearly identical RNAs that are subject to the same regulatory cues, as they will share the same promoter sequence and genomic neighborhood. While genes are transcribed from multiple different CTSSs, these CTSSs are grouped in a single or multiple TCs corresponding to the major RNAs (or transcripts/isoforms in RNA-Seq terminology) produced from the gene. Because of this, many studies use a simplification in which such TCs are referred to as TSSs for genes, even though technically TCs group several nearby bp-accurate TSS / CTSSs. To avoid confusion on terminology, and remain consistent with previous CAGE literature, we will primarily use the term `TC` to describe unidirectional clusters.

As an applied example, we analyzed three HeLa CAGE libraries from Andersson et al [47]. Using CAGEfightR, we calculated the pooled CTSS signal across libraries, and from that defined 22,760 TCs with > 1 TPM in at least two samples. As these TCs are defined de novo, it is useful to see how they relate to known transcript models. We therefore annotated the TCs according to University of California Santa Cruz (UCSC) transcripts using CAGEfightR’s hierarchical scheme (Fig. 2a). The hierarchical scheme accounts for the existence of multiple transcripts or isoform of genes, i.e. an annotated promoter of one transcript can be in the 5′-UTR of another transcript from the same gene. CAGEfightR can assign 9 different annotation categories (custom categories can be supplied by the user), based on the most likely association with known transcripts, e.g. a TC is more likely to correspond to an annotated promoter rather than a novel intragenic promoter.

Using this method, we plotted the number of TCs falling into the different annotation categories and their expression distributions (Fig. 2b). Most TCs candidates were found at annotated promoters and were generally highly expressed. However, a substantial number of novel (not overlapping annotated promoters) TCs were identified, in particular in the promoter-proximal region and 5′-UTR, which on average had lower expression than those overlapping annotated promoters.

In vertebrates, the distribution of CTSSs within TCs is related to cell specificity and DNA sequence properties: sharp CTSS distributions have an overrepresentation of TATA-boxes and are more cell- or tissue-specific, while broad CTSS distributions are GC-rich and more ubiquitously expressed [36]. Classification is often based on the width of the CTSS distribution, expressed as the interquartile/interquantile range (IQR), as this measures the width of the bulk of CTSSs within a TC without being affected by a few straggler CTSSs potentially greatly extending the width of the TC. We used CAGEfightR to calculate the genomic region covering the 5–95% IQR of total CTSS expression for each TC in the HeLa set (Similar results were obtained with tighter IQR intervals). This showed a clear bimodal distribution corresponding to sharp and broad CTSS distributions (Fig. 2c-d). Investigation of promoter regions using sequence logos also confirmed that sharp, but not broad distributions had a stronger TATA box (Fig. 2e).

### Analysis of enhancer candidates

Active enhancers are characterized by bidirectional transcription initiation of eRNAs [48]. In 5′-end data, this manifests as bidirectional clusters (BCs) of CTSSs, which can be used to systematically identify enhancer candidates [21]. Similarly to above, CAGEfightR uses a slice-reduce approach to identify bidirectional clusters (BCs, as opposed to the previously discussed unidirectional TCs) to predict enhancers (Fig. 1a, c). First, the upstream and downstream pooled CTSSs are quantified for every genomic position. Second, the Bhattacharyya coefficient [49] is used to quantify the departure of the observed pooled CTSS signal from perfect bidirectionality, producing a bidirectionality or balance score for each bp (Additional file 1: Figure S1A). Third, locations with a balance score above a given threshold are identified, and nearby sites are merged into discrete BCs. This slice-reduce approach is conceptually similar to the original enhancer prediction method by Andersson et al. [21], but does not need an input seed of TCs used to find bidirectional pairs, and gives similar results while being more scalable (Additional file 1: Figure S1B-C). As BCs can be found at other genomic regions than active enhancers (e.g. bidirectional gene promoters), BCs in or near known exons can be filtered away to obtain a final set of enhancer candidates [21].

As an applied example, we used the same CAGE HeLa data set as above and identified BCs outside of exonic regions and more than 1 kbp upstream of annotated promoters (based on UCSC transcript models) as enhancer candidates. This resulted in a total of 6384 enhancer candidates (3780 intronic and 2604 intergenic).

As an initial validation step, we investigated whether enhancer candidates had the expected chromatin patterns compared to TCs, by overlapping with DNase I hypersensitive sites sequencing (DNase-Seq), H3K27ac, H3K4me3 and H3K4me1 ChIP-Seq signals from the same cell type. As expected, we observed high DNase sensitivity at enhancer midpoints and TC peaks, and higher levels of H3K27ac at TCs, compared to enhancer candidates. The ratio of H3K4me3 to H3K4me1 is often used to predict enhancers from ChIP-Seq signals, and consistent with this we observed low average H3K4me3 and high H3K4me1 signals around predicted enhancer candidates, and the opposite patterns around TCs (Fig. 3a) [50, 51].

### Spatial prediction of enhancer-TSS links and enhancer clusters

An outstanding challenge in enhancer analysis is to link enhancers with target gene(s). Chromatin conformation capture data [52] is only available for a small set of cells, motivating computational prediction methods. A simple but popular linkage method is based on co-expression (correlation of expression) between enhancer candidates and genes across samples, assuming that true enhancer-gene pairs are co-expressed. This has been used for e.g. DNase and CAGE data, and serves as a reasonable hypothesis generator for physical interactions when the distance between enhancer and promoter is limited [21, 25, 53, 54]. CAGEfightR implements this approach by calculating the correlation of expression between enhancer candidates and TCs, with the option of supplying custom functions for calculating correlations in addition to the ones included in base R (Pearson, Spearman, Kendall).

As an example, we applied CAGEfightR to 50 CAGE samples obtained from colonic biopsies from ulcerative colitis and control subjects from Boyd et al. [25]. The reason for not using the HeLa set above was that correlations are more reliable if calculated across many samples. Specifically, we calculated a robust pooled CTSS signal (discarded CTSSs observed only in a single library), then selected TCs having > 1 TPM (and > 10% of total gene expression if they were assigned to genes, see below) in > 6 samples, and predicted enhancer candidates > = 1 CAGE tag in > 6 samples. This resulted in 31,480 TCs and 10,000 enhancer candidates. Using CAGEfightR, we computed the correlation (Kendall’s tau) between pairs of TCs and enhancer candidates within 10 kbp of each other, resulting in 19,271 positively correlated links between TCs and enhancers, where 978 were significant at FDR < 0.05. CAGEfightR supports the visualization of multiple enhancer candidate-TC links across a genomic region. As an example, Fig. 3b shows predicted enhancer-TC links around the TNFRSF1A gene, whose most dominant TC is most highly correlated with an intronic enhancer candidate in the neighboring gene.

Previously, large regions having enhancer-associated chromatin features were identified as drivers of central biological processes. Such regions are often referred to as “super”—or “stretch” enhancers [55]. Using CAGE data to predict enhancers, we have shown that many such chromatin-defined regions can be characterized as a group of bidirectionally transcribed loci, or a cluster of enhancer candidates [21, 25]. It follows that such larger regions can be predicted based on CAGE data, as genomic stretches with many enhancer candidates, and CAGEfightR implements methods for doing this. As an applied example, we used the enhancer candidates predicted based on the ulcerative colitis set above to identify enhancer clusters, defined as enhancers situated less than 12.5 kbp [25] from each other, resulting in 624 stretches with 4–24 enhancers per stretch. CAGEfightR can additionally calculate the average pairwise correlation between enhancer candidates in the stretch to reveal if they show concordant direction of change, indicative of joint activity, and provides methods to visualize these correlations (Fig. 3c).

### Analysis in terms of known gene models

Although TCs can be identified de novo, it is useful to be able to analyze their expression across known gene models. Examples include the ability to compare 5′-end expression with RNA-Seq expression on gene level [56], or to link 5′-end expression estimates with gene-centric databases, such as Gene Ontology (GO) terms [57] or pathway/interaction annotation (Kyoto Encyclopedia of Genes and Genomes (KEGG) [58], STRING [51], etc.). CAGEfightR includes functions for annotating TCs to known genes and summarizing their expression within genes to obtain a gene-level expression matrix (Fig. 1a, bottom). This gene-level expression matrix can readily be used with other Bioconductor packages for gene-level analysis (e.g. limma [44], edgeR [33], DESeq2 [35]).

Another key use of gene models in relation to 5′-end methods is the analysis of alternative TSS or alternative promoter usage, which is a key contributor in generating transcript diversity (multiple different transcripts/isoforms from genes). This can be done by identifying genes harbouring several TCs on the same strand, with each TC giving rise to distinct RNAs. In this way, TCs can be seen as TSS candidates for the different transcripts/isoforms produced by a gene, phrasing the analysis in a similar way to alternative splicing or transcript usage for RNA-Seq. To be clear, this is different from analyzing changes in the distribution of CTSSs *within* a TC (see above), as different TCs in a gene will be widely spaced, have different promoter sequences and genomic neighbourhoods and produce different truncations of RNA, with potentially different regulation and function.

In addition to identifying such alternative TCs within gene models, CAGEfightR offers the option of filtering TCs within genes based on their contribution to overall gene expression: As 5′-end methods can detect very lowly expressed TCs, CAGEfightR can remove alternative TCs making up less than e.g. 10% of total gene expression in a given number of samples to focus only on the most highly expressed RNAs from a gene. This filtering approach is also useful when combining CAGEfightR with popular tools for differential transcript usage such as limma [44], edgeR [33], DEXSeq [45] and DRIMSeq [46], to investigate whether a given TC within a gene is preferentially used under certain conditions.

To illustrate these features in CAGEfightR, we used the ulcerative colitis set, and assigned TCs to genes using UCSC gene models and determined how much each such TCs contributed to overall gene expression. Without any composition filtering, 40% of all genes used more than one TC, falling to 23% when only considering TCs contributing more than 10% of total gene expression in more than 6 samples (Fig. 4a). The majority of discarded TCs were found in protein-coding, intronic and 3′-UTR regions (Fig. 4b), but several interesting cases remained, for example in the SLC16A5 gene, where we identified a highly expressed novel intronic TC (Fig. 4c).

### Example of PRO-cap data analysis using CAGEfightR

While conceived as a tool for analyzing CAGE data, CAGEfightR can analyze any 5′-end data similar to CAGE, including nascent RNA 5′-end methods (Table 1). This is highly relevant since nascent 5′-end methods may be more sensitive in terms of enhancer detection, and hence are often used specifically for this purpose. To illustrate the usefulness of CAGEfightR for analyzing such data, we applied CAGEfightR to 59 lymphoblastoid cell line Precision Nuclear Run-on Sequencing for RNA Polymerase II Start Sites (PRO-Cap) libraries from Katla et al [59]. Similarly to the analysis of ulcerative colitis CAGE data above, robust pooled CTSSs (CTSSs observed in > 2 samples) were used to identify TCs (> 1 TPM in > 5 samples) and enhancer candidates (> 0 tags in > 5 samples), and annotated these using UCSC transcript models. Compared to the CAGE libraries above, a larger number of antisense TCs, intergenic TCs and enhancer candidates were detected (Fig. 5a). This is expected as these RNAs are subject to nuclear degradation and thus are more difficult to detect with steady-state RNA methods. PRO-Cap TCs showed the expected pyrimidine-purine di-nucleotide at positions − 1 + 1 (Fig. 5b), and enhancer candidates exhibited the characteristic H3K4me3 to H3K4me1 ratio (Fig. 5c). Figure 5d shows an example of an enhancer candidate detected by PRO-Cap.

## Conclusions

CAGEfightR is a user-friendly R-Bioconductor package for analyzing CAGE and 5′-end data. It includes a wide range of functions for analyzing TSSs and enhancers and generating publication-ready visualizations. CAGEfightR was designed from the ground up to adhere closely to Bioconductor standards, making it easy to learn, use and combine CAGEfightR with other Bioconductor packages for transcriptomic analyses. CAGEfightR is extensively documented, including both a vignette describing the core functionality (http://bioconductor.org/packages/CAGEfightR/) and a case-study based workflow discussing common analysis tasks on a full dataset [60] (http://bioconductor.org/packages/CAGEWorkflow/).

## Materials and methods

### Datasets

Analysis of all 5′-end data was based on supplied CTSSs from the GEO repositories of the respective papers. The Hela set [47] was obtained from *GSE62047*, using only EGFP samples. The ulcerative colitis set [25] was obtained from *GSE95437*, using only active ulcerative colitis (UCa) samples from the largest batch. The PRO-Cap set [59] was obtained from *GSE110638*, using only non-replicated samples.

Transcript and gene models were obtained from UCSC via the R/Bioconductor packages *TxDb.Hsapiens.UCSC.hg19.knownGene* and *TxDb.Mmusculus.UCSC.mm9.knownGene*. Chromatin data was obtained from the Roadmap Epigenomics project [61] via the *AnnotationHub* R/Bioconductor package, for Hela cells AH32877, AH32879, AH32881 and AH32884 and for Lymphoblastoid cell lines AH32865, AH33899, AH33901 and AH33904.

### Analysis

All analyses of 5′-end data were done using CAGEfightR as indicated in the main text. Average meta profiles were made using the TeMPO R-package (https://github.com/MalteThodberg/TeMPO), removing the top 1% highest scoring features to dampen the effect of outliers. Sequence logos were done using the ggseqlogo R-package [62], genome-browser figures using Gviz [42] and remaining figures using ggplot2 (https://www.tidyverse.org/).

Andersson enhancers were predicted using scripts from the original publication [21] (https://github.com/anderssonrobin/enhancers). TCs used as input were defined by CAGEfightR with default settings. A balance cutoff of 0.6 was used, as this corresponds to the 0.95 balance cutoff used in CAGEfightR in the case of a BC with only divergent signal (PD and MD in Additional file 1: Figure S1A).

## Availability and requirements

**Project name:** CAGEfightR.

**Project home page:** https://bioconductor.org/packages/release/bioc/html/CAGEfightR.html

**Operating system(s):** Platform independent (BigWig I/O only available on Windows).

**Programming language:** R.

**Other requirements:** Bioconductor.

**License:** GPL-3.

Any restrictions to use by non-academics: GPL-3.

## Additional file

Additional file 1: **Figure S1.** Details on finding Bidirectional Clusters (BCs). A: Calculating balance score using the Bhattacharyya coefficient. For a potential BC midpoint, pooled CTSS signal is summed within a certain distance (200 bp by default) on both strands, yielding four values (left). The “ideal” bidirectional cluster would have only perfect divergent signal (50% PD and 50% MD). The Bhattacharyya Coefficient quantifies the difference between the observed signal to this ideal enhancer (right), with a balance score of 1 indicating perfect agreement. The balance score is calculated for every bp in the genome (Fig. 1c). B: Overlap between CAGEfightR and Andersson enhancer predictions. The original enhancer prediction method from Andersson et al were applied to the Hela set. The venn diagram shows overlap in predictions between CAGEfightR, Andersson et al and DNase hypersensitive sites. CAGEfightR predicts all enhancers candidates form Andersson et al that are also supported by DNase hypersensitive sites. C: Chromatin modifications at CAGEfightR and Andersson predicted enhancer from the Hela set. X-axis shows distance to enhancer midpoint. Y-axis are average signal of respective DNase-Seq or ChIP-Seq data in the given panel row. Color indicates the enhancer candidate sets (Andersson in gold and CAGEfightR in grey), with panel columns indicating whether enhancer are shared between sets (left) or uniquely predicted (right). All sets exhibits the characteristic DNase hypersensitivity and H3K4me1/H3K4me3 ratio, despite the CAGEfightR enhancer candidate set is much larger. (PDF 840 kb)

## Data Availability

The CAGEfightR R/Bioconductor package is freely available from http://bioconductor.org/packages/CAGEfightR/. All analyses are based on publicly available data obtained via GEO and AnnotationHub (See Materials and Methods for accession numbers).

## Abbreviations

5′−/3′-UTR: 5 prime / 3 prime untranslated region
BC: Bidirectional Cluster
bp / kbp: Base pair / kilo base pairs.
CAGE: Cap Analysis of Gene Expression
CDS: Coding sequencing
ChIP-Seq: Chromatin Immunoprecipitation Sequencing
CTSSs: CAGE Transcription Start Sites
DNase-Seq: DNase I hypersensitive sites sequencing
GO: Gene Ontology
IQR: Interquartile/Interquantile Range
KEGG: Kyoto Encyclopedia of Genes and Genomes
PRO-Cap: Precision Nuclear Run-on Sequencing for RNA Polymerase II Start Sites
RNA-Seq: RNA-Sequencing
TC: Tag Cluster or Unidirectional Cluster (closely spaced array of tags on the same strand)
TPM: Tags-Per-Million
TSS: Transcription Start Site
UCSC: University of California Santa Cruz
