# Supplementary material for: CAGEfightR: analysis of 5′-end data using R/Bioconductor
Source: BMC Bioinformatics. 2019 Oct 4;20:487. doi: 10.1186/s12859-019-3029-5 (PMC6778389; doi:10.1186/s12859-019-3029-5)
Supplement: Supplementary file 1 — Figure S1. Details on finding Bidirectional Clusters (BCs). A: Calculating balance score using the Bhattacharyya coefficient. For a potential BC midpoint, pooled CTSS signal is summed within a certain distance (200 bp by default) on both strands, yielding four values (left). The “ideal” bidirectional cluster would have only perfect divergent signal (50% PD and 50% MD). The Bhattacharyya Coefficient quantifies the difference between the observed signal to this ideal enhancer (right), with a balance score of 1 indicating perfect agreement. The balance score is calculated for every bp in the genome (Fig. 1c). B: Overlap between CAGEfightR and Andersson enhancer predictions. The original enhancer prediction method from Andersson et al were applied to the Hela set. The venn diagram shows overlap in predictions between CAGEfightR, Andersson et al and DNase hypersensitive sites. CAGEfightR predicts all enhancers candidates form Andersson et al that are also supported by DNase hypersensitive sites. C: Chromatin modifications at CAGEfightR and Andersson predicted enhancer from the Hela set. X-axis shows distance to enhancer midpoint. Y-axis are average signal of respective DNase-Seq or ChIP-Seq data in the given panel row. Color indicates the enhancer candidate sets (Andersson in gold and CAGEfightR in grey), with panel columns indicating whether enhancer are shared between sets (left) or uniquely predicted (right). All sets exhibits the characteristic DNase hypersensitivity and H3K4me1/H3K4me3 ratio, despite the CAGEfightR enhancer candidate set is much larger. (PDF 840 kb) [file 12859_2019_3029_MOESM1_ESM.pdf]

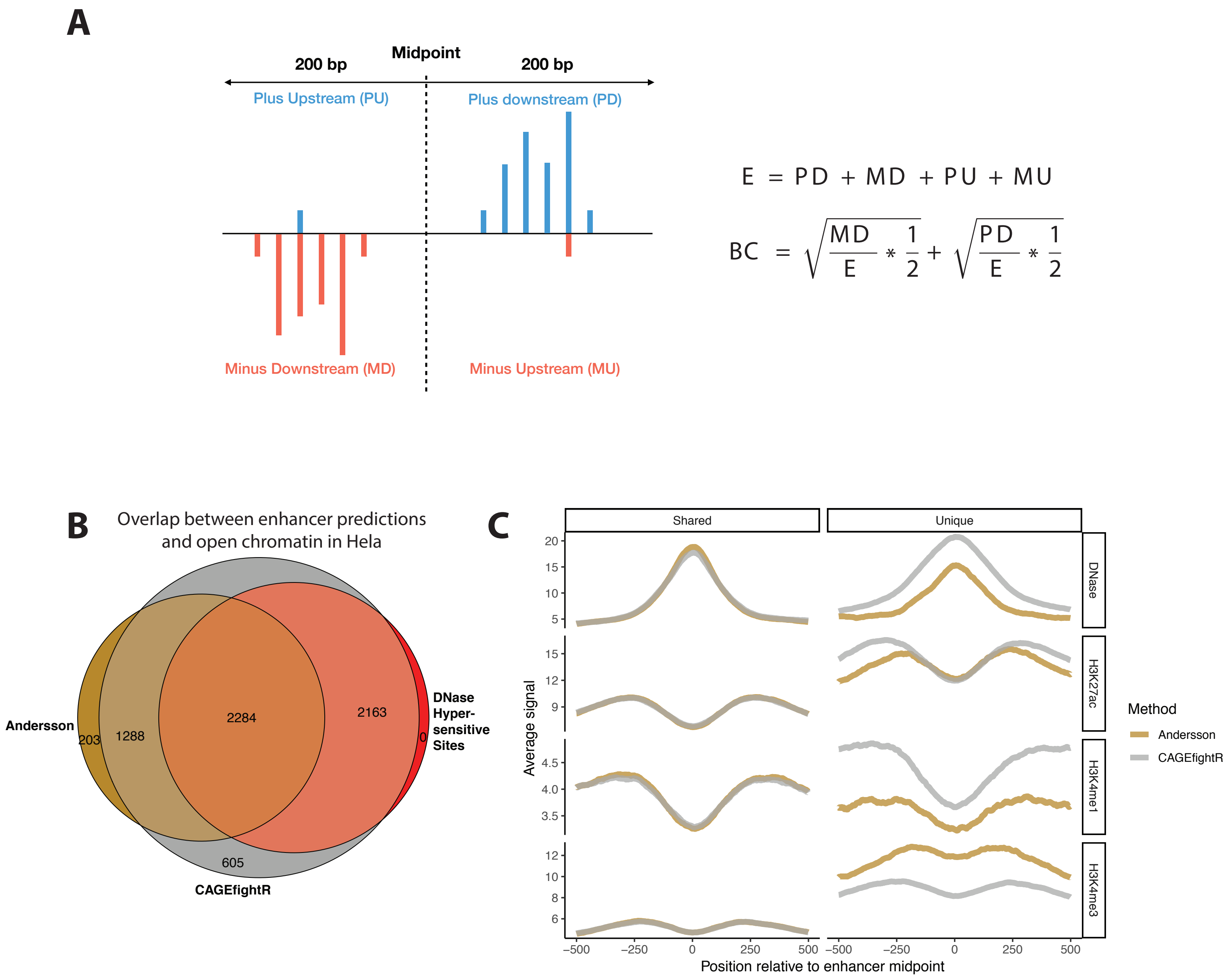
A
Midpoint
200 bp
200 bp
Plus Upstream (PU)
Plus downstream (PD)
Minus Downstream (MD)
Minus Upstream (MU)
E = PD + MD + PU + MU
BC = √(MD/E * 1/2) + √(PD/E * 1/2)
B
Overlap between enhancer predictions and open chromatin in Hela
Andersson
203
1288
2284
2163
0
DNase Hyper-sensitive Sites
605
CAGEfightR
C
Shared
Unique
DNase
H3K27ac
H3K4me1
H3K4me3
Average signal
Position relative to enhancer midpoint
Method
Andersson
CAGEfightR
